# The (cost) effectiveness of a very low-energy diet intervention with the use of eHealth in patients with type 2 diabetes and obesity: study protocol for a randomised controlled non-inferiority trial (E-diet trial)

**DOI:** 10.1186/s13063-023-07620-6

**Published:** 2023-10-05

**Authors:** Karlijn A. M. Geurts, Behiye Ozcan, Mandy van Hoek, Roel van de Laar, Jolande van Teeffelen, Joost van Rosmalen, Elisabeth F. C. van Rossum, Kirsten A. Berk

**Affiliations:** 1 Department of Internal Medicine, Division of Dietetics, Erasmus MC, University Medical Center, Rotterdam, The Netherlands; 2 Department of Internal Medicine, Division of Diabetology and Division of Vascular Medicine, Erasmus MC, University Medical Center, Rotterdam, The Netherlands; 3 Department of Internal Medicine, Ikazia Hospital, Montessoriweg 1, 3083 AN Rotterdam, The Netherlands; 4 Dietician Practice Health Risk Control, Henk Speksnijderstraat 27, 3067 AC Rotterdam, The Netherlands; 5 Department of Biostatistics, Erasmus MC, University Medical Center, Rotterdam, The Netherlands; 6 Department of Epidemiology, Erasmus MC, University Medical Center, Rotterdam, The Netherlands; 7 Department of Internal Medicine, Division of Endocrinology, Erasmus MC, University Medical Center, Rotterdam, The Netherlands; 8 Division of Endocrinology, Obesity Center CGG, Erasmus MC, University Medical Center, Rotterdam, The Netherlands

**Keywords:** Type 2 diabetes, Overweight, Obesity, Diet, Very low energy diet, eHealth, Mobile application, Blended care, Non-inferiority

## Abstract

**Background:**

Despite preventive measures, the number of people with type 2 diabetes and obesity is increasing. Obesity increases morbidity and mortality in people with type 2 diabetes, making weight loss a cornerstone of treatment. We previously developed a very low energy diet (VLED) intervention that effectively reduced weight in people with type 2 diabetes in the long term. However, this intervention requires considerable time and manpower, which reduces the number of people who can benefit from it. eHealth offers more efficient solutions but has proven to be less effective than face-to-face interventions. Therefore, we want to investigate whether a blended version of our VLED intervention (in which face-to-face contact is partly replaced by an eHealth (mobile) application (E-VLED)) would be more cost-effective than the current face-to-face intervention.

**Methods:**

We will conduct a randomised, controlled trial with non-inferiority design in patients with type 2 diabetes and obesity (BMI > 30 kg/m^2^), aged 18–75 years. The control group will receive the usual care VLED intervention, while the intervention group will receive the E-VLED intervention for 1 year, where face-to-face contact will be partly replaced by an eHealth (mobile) application. The main study endpoint is the difference in weight (% change) between the control and intervention group after 1 year, plus the difference between the total costs (euro) of the treatment in the control and intervention groups. The secondary aims are to investigate the effectiveness of the E-VLED diet intervention regarding cardiovascular risk factors, quality of life, patient satisfaction, compliance, and to study whether there is a difference in effectiveness in pre-specified subgroups. General linear models for repeated measurements will be applied for the statistical analysis of the data.

**Discussion:**

We hypothesise that the E-VLED intervention will be equally effective compared to the usual care VLED but lower in costs due to less time invested by the dietician. This will enable to help more people with type 2 diabetes and obesity to effectively lose weight and improve their health-related quality of life.

**Trial registration:**

Netherlands Trial Register, NL7832, registered on 26 June 2019.

## Administrative information

**Table:** 

Title {1}	The (cost) effectiveness of a very low energy diet intervention with or without the use of eHealth in obese patients with type 2 diabetes: a randomised controlled non-inferiority trial (E-diet trial}
Trial registration {2a and 2b}	Netherlands Trial Register, NL7832, registered on 26 June 2019
Protocol version {3}	Version 4, date 12–11-2020
Funding {4}	Erasmus MC, Mrace efficiency research
Author details {5a}	1 Department of Internal Medicine, division of Dietetics, Erasmus MC, University Medical Center, Rotterdam, Doctor Molewaterplein 40, 3015 GD Rotterdam, The Netherlands 2 Department of Internal Medicine, division of diabetology and division of Vascular Medicine, Erasmus MC, University Medical Center, Rotterdam, Rotterdam, The Netherlands 3 Department of Internal Medicine, Ikazia hospital, Montessoriweg 1, 3083 AN Rotterdam, The Netherlands 4 Dietician Practice Health risk control, Henk Speksnijderstraat 27, 3067 AC Rotterdam, The Netherlands 5 Department of Biostatistics, Erasmus MC, University Medical Center, Rotterdam, Rotterdam, The Netherlands 6 Department of Internal Medicine, Division of Endocrinology, Erasmus MC, University Medical Center Rotterdam, Rotterdam, The Netherlands 7 Obesity Center CGG, Division of Endocrinology, Erasmus MC, University Medical Center Rotterdam, Rotterdam, The Netherlands 8 Department of Epidemiology, Erasmus MC, University Medical Center Rotterdam, Rotterdam, The Netherlands
Name and contact information for the trial sponsor {5b}	Erasmus MC Dr. Molewaterplein 40 3015 CE Rotterdam
Role of sponsor {5c}	The sponsor provided the necessities for conducting the research. It had no role in study design; collection, management, analysis, and interpretation of data; writing of the report; or the decision to submit the report for publication

## Introduction

### Background and rationale {6a}

Type 2 diabetes (T2D) affects 422 million adults worldwide, of whom approximately 85% are living with overweight or obesity. In the Netherlands, over one million people already have T2D, and it has been estimated that one out of every three Dutch adults will develop T2D in his or her life [1, 2]. When a person has the combination of both T2D and obesity, he or she will have more complications and an increased risk of cardiovascular disease and mortality [3].

Weight loss alleviates this problem and improves cardiovascular risk profile and quality of life and reduces the risk of more than 200 other obesity-related diseases [4]. Our previous study showed that a diet program based on a very low energy diet (VLED) led to long-term weight loss (2 years), improved quality of life, improvement in depressive symptoms, and a lower need for insulin in patients who have had T2D for a long time [5]. A VLED is a diet that contains 400–800 kcal per day, with two meal replacements with adequate protein intake [6]. An evaluation of the program demonstrated that 95% of participants would recommend the program to others. The DiRECT trial showed similar results for a VLED intervention after 2 years in patients with recently diagnosed T2D: a decrease in mean body weight and improved quality of life for patients in the intervention group [7].

However, sustainable weight loss is difficult to achieve without professional help, and with the growing rates of T2D and obesity, the pressure on our health care system increases in terms of morbidity and costs [8]. In order to be successful, lifestyle intervention programs need to be of high intensity (with frequent contacts) and multidisciplinary, making them costly and limited to available human resources [9, 10].

eHealth-based treatments are a promising tool to provide dietary support at a lower cost than face-to-face interventions, especially when extended follow-up is needed. The term eHealth, is defined by Eysenbach et al. “eHealth refers to health services and information delivered or enhanced through the Internet and related technologies. Moreover, eHealth applications may increase access, improve convenience, and increase participant engagement [11–13]. However, eHealth interventions seem less effective in producing weight loss than face-to-face “in-person” treatment [14–16]. Among people with T2D, there is a great deal of variation in the type of eHealth used in dietary interventions. In general, the use of eHealth alone is less effective in reducing weight. Combining a diet program with the use of some form of eHealth is more effective in terms of weight loss.

Hypothetically, blended care with the combination of in-person treatment and eHealth solutions, is the most (cost)effective [12]. However, the (cost)effectiveness of an E-VLED weight loss intervention in people with T2D has not been studied to date.

## Objectives {7}

The primary objectives of this study are as follows:

1. To determine if an E-VLED intervention is equally effective as the usual care VLED in reducing weight after one year in people with T2D and obesity, and
2. To determine if an E-VLED intervention is lower in costs than the usual care VLED.

The secondary objectives of this study are as follows:

1. To determine the effectiveness of an E-VLED intervention regarding cardiovascular risk factors and quality of life, compared to a VLED intervention
2. To determine the effectiveness of an E-VLED intervention regarding patient satisfaction and attrition/compliance, compared to a VLED intervention
3. To determine if there is a difference in effectiveness and patient satisfaction of an E-VLED intervention between males and females
4. To determine if there is a difference in effectiveness and patient satisfaction of an E-VLED intervention between participants of different origins

### Trial design {8}

**Fig. 1:**
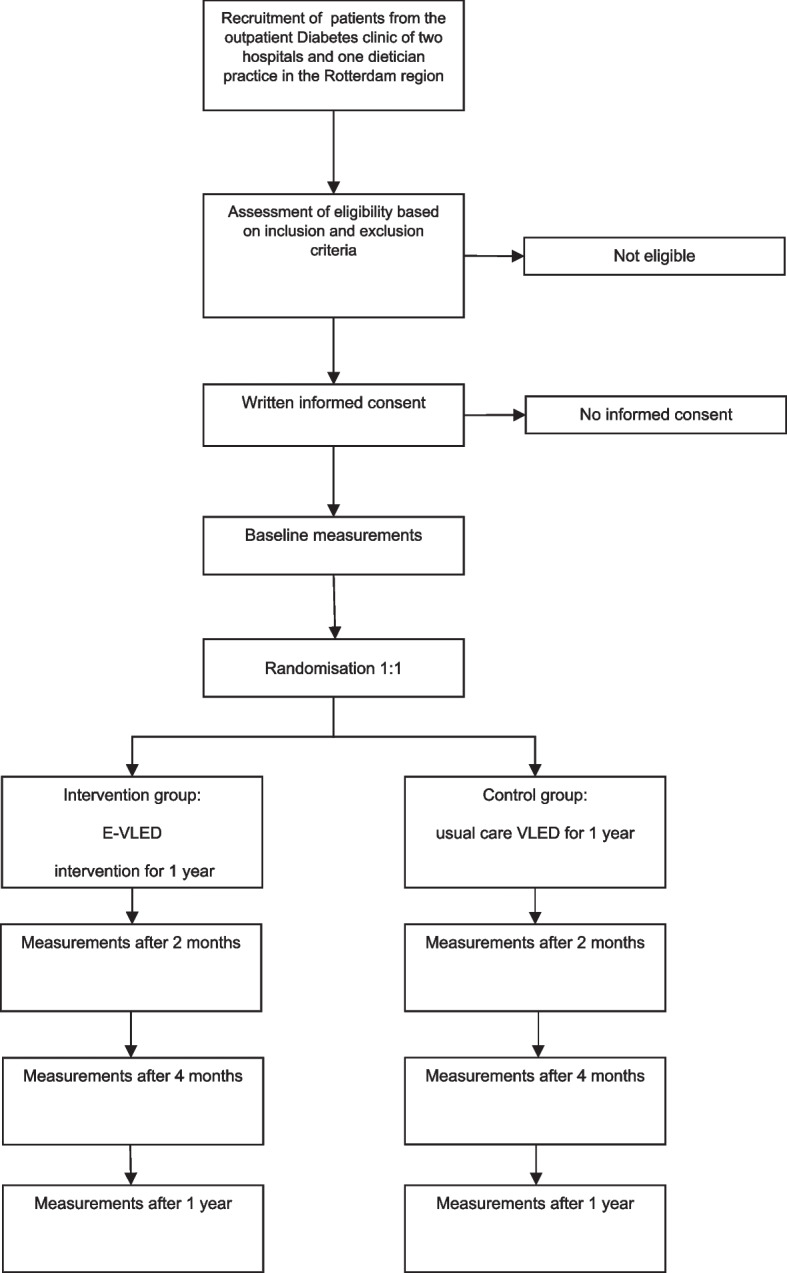
Flow chart of the trial

This study is a randomised, controlled, non-inferiority multi-centre trial with a duration of 1 year. After signing informed consent, eligible participants will be randomised 1:1 to the intervention (E-VLED) or control (VLED) group. Outcome parameters will be measured at baseline, 2 months, 4 months, and at 1 year. In Fig. 1, we show the flow chart of this study.

## Methods: participants, interventions, and outcomes

### Study setting {9}

Patients with T2D and obesity will be recruited from the Diabetes outpatient clinic of the University Medical centre, Erasmus MC, of the Ikazia hospital, and dietician practice HRC, all located in Rotterdam, The Netherlands.

### Eligibility criteria {10}

Inclusion criteria.

- Type 2 diabetes
- Age 18–75 years
- Overweight/obesity (BMI ≥ 27 kg/m.^2^)
- Smartphone with Android or iOS

Exclusion criteria.

- Pregnancy or lactation during the study
- Insufficient command of the Dutch language, spoken or written assessed in the intake interview
- Severe psychiatric problems, use of antipsychotic drugs
- Significant cardiac arrhythmias; unstable angina; decompensated congestive heart failure; carcinomas; major organ system failure; untreated hypothyroidism; end-stage renal disease
- Myocardial infarction, cerebrovascular accident, or major surgery during the previous 3 months
- Other types of diabetes
- Substantial wounds like diabetic foot
- Using glucagonlike peptide-1 (GLP-1) medication for < 3 months

The appointments and intervention will be performed by a researcher/dietician specialised in diabetes and lifestyle.

### Who will take informed consent? {26a}

Patients will be given at least two weeks to decide whether they want to participate after they receive the information by the research coordinator. Inclusion is complete after the participant and the researcher sign the informed consent form.

### Additional consent provisions for collection and use of participant data and biological specimens {26b}

We ask for informed consent for the collection of one tube of blood for biobanking, in order to answer future research questions.

### Interventions

#### Explanation for the choice of comparators {6b}

In people with T2D and overweight/obesity, the use of a VLED intervention has been shown to be effective in achieving weight reduction and reducing cardiovascular risk. However, the current method of counselling is very labour-intensive and time-consuming and limited to people who can regularly visit the hospital. By using eHealth, this problem may be solved. Therefore, we want to compare a VLED in its current face-to-face counselling form with the same VLED in which part of the face-to-face contacts are replaced using a mobile application.

### Intervention description {11a}

#### The control and intervention group

Both groups will receive a VLED, with meal replacements (The 1:1 diet by Cambridge Weight Plan®), plus a stepped care approach to reintroduce a healthy and sustainable long-term diet according to national guidelines [17]. Patients will follow a VLED of approximately 3140 kJ (750 kcal)/day for 10 weeks, consisting of two meal replacements plus 75 g of lean meat/fish/vegetarian replacement, 150 ml skimmed milk, and low-energy drinks and low-carbohydrate vegetables ad libitum.

After 10 weeks, the diet is changed into a low-calorie diet of 4600–5400 kJ (1100–1300 kcal)/day, gradually increasing the intake during the following 12 weeks. After a total of 22 weeks, the participants use a diet based on national health recommendations, aimed at weight maintenance. During the entire intervention, 60 min of exercise per day is advised. We encourage long-term behavioural change by making use of behavioural therapy techniques (among others: goal setting, identifying barriers and self-monitoring). During the diet, glucose levels (fasted and postprandial) are monitored (via phone) and glucose lowering agents are adjusted accordingly, by the dieticians, diabetes nurses, and medical doctor. Moreover, antihypertensive medication is adjusted whenever the blood pressure becomes too low due to the weight loss. The process is guided by registered dieticians of the University Medical centre, Erasmus MC in Rotterdam, and dietician practice HRC.

#### The intervention group

The eHealth application, which replaces part of the face-to-face contacts, will only be used by the intervention group. In-person contact will be limited to one individual intake meeting and three group meetings. The eHealth application is a mobile diet app, which was developed earlier and has already been pilot tested. In this app, the steps of the diet-intervention will be explained via text and infographics. Participants will receive recipes and tips useful for the different stages of the diet. Exercise is encouraged with personal goal setting, using the national guidelines for exercise [18], and challenges. Personal goal setting with respect to diet, exercise, weight, body composition, glucose levels, medication use, physical complaints, and quality of life will be used and monitored within the app. Moreover, participants will receive daily motivational messages to keep them on-track. To make the app more interactive, the participants can use a chat-function with the dietician, once a week for the first 10 weeks; thereafter, the dietitian will be available every other week for the chat feature in the app. The app is end-to-end encrypted, according to the national privacy guidelines.

#### The control group

In this group, the participants will follow the VLED intervention and will receive one individual intake meeting, five group meetings, and four planned contact moments by mail or phone (more whenever it is necessary for glucose regulation). In this group, the motivational aspect and personal goal setting with respect to diet, exercise, weight, body composition, glucose levels, medication use, physical complaints, and quality of life will be monitored during the group meetings.

### Criteria for discontinuing or modifying allocated interventions {11b}

Subjects can leave the study at any time for any reason if they wish to do so, without any consequences. The investigator can decide to withdraw a subject from the study for urgent medical reasons. We will analyse the study results according to the intention-to-treat principle; withdrawn participants will not be replaced.

Patients who withdraw from the study will be asked (voluntarily) about their reasons for withdrawal and will receive the usual treatment of the diabetes medical team.

### Strategies to improve adherence to interventions {11c}

Patients in the intervention group will receive, throughout the year, notifications in the app to promote their adherence to the intervention.

The control group will receive a phone call 1 week after the group sessions to promote adherence to the intervention.

Both groups will receive a book on healthy nutrition and lifestyle developed by the Dutch cooperation of dieticians as an incentive. This book provides additional information on a healthy lifestyle beyond the group sessions and the information in the app.

### Relevant concomitant care permitted or prohibited during the trial {11d}

Patients will receive their usual medical care and are allowed to use all their prescribed medication. Anti-diabetic medication will be adjusted before the start of the intervention, according to the guideline demedicalisation for T2D [19]. Furthermore, medication is individually adjusted when hypo- or hyperglycaemia do often occur. Other medications such as antihypertensive medication will be adjusted as required and always in agreement with the medical team. No concomitant care will be prohibited during the study.

### Provisions for post-trial care {30}

After finishing the study, patients will continue receiving their usual diabetes treatment, including dietary treatment.

### Outcomes {12}

Main study parameters are as follows:

- Difference in weight (% change) between the control and intervention group after 1 year. Weight will be measured, after removal of shoes using a Seca 888 compact digital flat scale
- Difference in costs will be determined by the difference in costs of the treatment, as well as costs of health care and costs of production losses, measured by the Trimbos/iMTA Questionnaire for Costs (TiC-P) [20]

Secondary study parameters are as follows:

- Quality of life, measured with the EuroQol questionnaire EQ-5D-5L [21]
- Diabetes regulation measured by HbA1c (mmol/mol) via routine laboratory procedures

Other study parameters are as follows:

- Cardiovascular risk factors: blood pressure (mmHg), total cholesterol (mmol/l), LDL cholesterol (mmol/l), HDL cholesterol (mmol/l), triglycerides (mmol/l), fasting blood glucose (mmol/l), all measured via routine clinical care/lab

### Participant timeline {13}

The participant timeline is presented in Table 1.

**Table 1:** Schedule of enrolment, interventions, and assessments

	**Study period**						
	**Enrolment**	**Allocation**	**Post-allocation**				**Close-out**
**Timepoint**	***-T***_***1***_	**0**	***T***_***0***_ *Start*	***T***_***1***_ *Week 10*	***T***_***2***_ *Week 20*	***T***_***3***_ *Week 30*	***T***_***4***_ *Week 52*
**Enrolment:**							
Eligibility screen	X						
Informed consent	X						
Discuss in multidisciplinary consultation	X						
Allocation		X					
**Study visits:**							
E-VLED			X		X		X
VLED			X	X	X	X	X
**Assessments:**							
Laboratory measurement venipuncture			X	X	X		X
Biobank vena punction			X	X	X		X
Length			X				
Waist circumference			X	X	X		X
Body composition			X	X			X
Resting energy expenditure			X	X			X
Dietary history			X	X			X
Physical activity			X	X	X		X
Questionnaire about demographic variables, lifestyle and medication use			X	X	X		X
EQ-5D-5L			X	X	X		X
DTSQ			X	X	X		X
EHIQ			X	X	X		X
HADS			X	X	X		X
Weight			X	X	X		X
TiC-P			X	X	X		X

### Sample size {14}

In our previous study using the same diet intervention [5], the standard deviation of the difference in weight loss at 12 months was 7.58 kg, and the baseline weight was 105 kg; thus, the standard deviation percentage weight loss was 7.2%. In the field of long-term weight loss, two weight loss percentages are considered clinically relevant, 3% and 5% [22–24]. In order to stay on the safe side, we choose the non-inferiority margin based on the lower of these two percentages, i.e. a non-inferiority margin of 3%. Results of recently published randomised controlled trials on dietary eHealth interventions [25, 26] have yielded estimates for the percentage weight loss due to these interventions. In light of these results, we assumed in the power analysis that the weight loss percentage is 2% greater in the intervention arm than in the control arm. This estimate of 2% was obtained by averaging the results of Katula et al. (− 3.4%) and Baer et al. (1.1%). Based on a one-sided alpha of 0.025, power set at 0.80, we calculated that at least 33 participants in each study group are required for the present study (calculated using R). Anticipating a drop-out of 20%, based on attrition data of the POWER study, we aim for the inclusion of 80 participants (40 participants per group).

### Recruitment {15}

Patients will be recruited from the outpatient diabetes clinic of the University Medical centre, Erasmus MC in Rotterdam, the Ikazia Hospital in Rotterdam and dietician practice HRC in Rotterdam by the researcher, based on the inclusion and exclusion criteria. Patients interested in participating in the study will receive the patient information letter by the coordinating researcher. If the patient is still interested in participating, a telephone consultation is scheduled in which the investigator will check eligibility and patients will have the opportunity to ask their questions about the study. After the patient signs the informed consent form, the appointment for the baseline measurements will be set up, and the participant will be allocated to the control or intervention group, and receive information on the first kick-off group meeting.

### Assignment of interventions: allocation

#### Sequence generation {16a}

Eligible patients who signed informed consent will be randomly assigned to either the intervention group or the control group in groups of ten patients. This means that patients who have given informed consent will be randomised only when a group of ten patients is reached, who will then all be in the same treatment group. The 8 groups of ten patients will be randomised with an allocation ratio of 1:1, using blocked randomisation with stratification by centre. The initial block size per centre is two groups (i.e. 10 patients per randomisation arm), and in two of the three centres, there will be a second block of one group. The randomisation, according to a random block design stratified to study site, is supervised by JR and is blinded for the researcher/dietician recruiting and/or performing the intervention. The randomisation is computer controlled.

### Concealment mechanism {16b}

We will make use of a central telephone randomisation system, where the allocation sequence is only available to the secretariat of the Department of Dietetics. The investigator will contact the secretariat by phone, when a group of ten patients is complete and has signed the informed consent forms, to receive the allocation of the group.

### Implementation {16c}

The allocation sequence generated by the statistician will be kept in a secure place in the dietetics department secretariat of the different study sites, where only the secretary has access to the randomisation list. When a group of ten patients is complete, the researcher will contact the secretary of that study site by telephone to obtain the allocation, after which the patients of that group will be enrolled by the researcher.

### Assignment of interventions: blinding

#### Who will be blinded {17a}

Given the nature of the intervention, the participants could not be blinded. Due to practical reasons, we elected not to blind investigators and medical staff. However, the first preliminary statistical analyses of the primary outcome will be performed by an independent statistician, blinded to the allocation.

### Procedure for unblinding if needed {17b}

The design is open label with only data analysts being blinded, so un-blinding will not occur.

### Data collection and management

#### Plans for assessment and collection of outcomes {18a}

Outcome data will be collected in a case report form designed in a data management system (Castor EDC®). Questionnaires will be sent digitally to participants using Castor EDC®. To promote data quality of the measurements, all researchers will be trained to perform the measurements. To ensure the data quality of the questionnaires, these will be checked by the researcher to see if there are any irregularities.

Study instruments used:

- Weight will be measured using a Seca 888 compact digital flat scale
- Costs will be measured by the Trimbos/iMTA Questionnaire for Costs (TiC-P)
- Quality of life will be measured with the EuroQol questionnaire EQ-5D-5L
- Blood pressure will be measured using the Omron, M4-I
- Food intake will be measured using dietary history [27]
- Physical activity will be measured by wearing an Activ8 sensor (BV, Valkenswaard, The Netherlands) for 1 week. The sensor will measure non-wear, sitting, standing, walking, cycling, or running in counts per 20 s
- Waist circumference will be measured with a centimetre
- Body composition will be measured with the Bodystat Quadscan 4000, Euromedix, Leuven, Belgium
- Resting energy expenditure will be measured using the Q-NRG, Cosmed Benelux B.V., Nieuwegein, The Netherlands. Patient satisfaction will be measured via the Diabetes Treatment Satisfaction Questionnaire (DTSQ) [28, 29]
- Attitudes towards using eHealth (general and specific for this diet app) to access health information, measured via the eHealth Impact Questionnaire (EHIQ) [30]
- Depression and anxiety via the Hospital Anxiety and Depression Scale (HADS) [31]
- Adherence will be measured with log-on information of the app
- Compliance with the diet will be measured by analysing dietary history (energy intake compared to advised energy intake in %)
- Attrition will be measured by the number of participants that drop-out (categorised by reason for drop-out). Practical use of the intervention diet-app, via self-developed questionnaire, focus groups, and the System Usability Scale (SUS) [32]
- Demographic variables, drug use, smoking and drinking habits, exercise, and medication use will be measured using a self-developed questionnaire.

### Plans to promote participant retention and complete follow-up {18b}

Patients will receive a notification in the app (intervention group) or via mail (control group) of research appointments. They will also be notified if questionnaires are not completed on time. Also, when patients are not active in the app for the first 1 to 2 weeks, the patient is contacted to see if there is an issue with the app that can be resolved. When patients do not show up for their appointments, they will be contacted by phone to find out the reason for their absence. Their appointment will be rescheduled if possible.

### Data management {19}

Data will be entered into a data management system (Castor EDC®, Amsterdam, The Netherlands) with a consecutive code number (no initials or date of birth). All original paperwork, including informed consent, will be kept in a locked room/closet at the primary study site. All data, digital and on paper, will be kept for a maximum of 15 years after the study has finished.

### Confidentiality {27}

The subject identification code list, which links the code number to the participant, will be safeguarded by the coordinating researcher in a ‘master file’. The key to the code is only accessible by the investigators. Study data can only be accessed by the investigator team, staff of the Health Care Inspection and members of the Medical Ethical Committee, as stated in the informed consent form. The handling of personal data will comply with EU General Data Protection Regulation and the Dutch Act on Implementation of the General Data Protection Regulation.

### Plans for collection, laboratory evaluation, and storage of biological specimens for genetic or molecular analysis in this trial/future use {33}

We will collect samples of fasted blood (serum and plasma) for study purposes only. The other laboratory measurements are part of routine care. These blood samples will be encoded and stored at − 80 °C until analysis. All blood samples will be kept for a maximum of 15 years after the study has finished.

### Statistical methods

#### Statistical methods for primary and secondary outcomes {20a}

All analyses will be conducted according to the intention-to-treat principle. Normality of the data and homogeneity of variances will be tested using Shapiro–Wilk test and Levene’s test. As measures of central tendency for numerical data, we will use the mean (in case of normal distribution) and median values (in case of non-normal distribution), with respectively the standard deviation and interquartile range as measures of dispersion.

The primary endpoint will be defined as the between group difference in relative weight change during 12 months. The relative weight change will be analysed with a general linear model for repeated measurements, with independent variables treatment arm, time point (T1, T2, or T3), hospital, and the interaction between treatment arm and time point. The within patient correlations will be modelled using an unstructured covariance matrix. The estimated difference between treatment arms (intervention minus control) in the relative weight change at T3 (12 months) will be calculated using the estimated marginal means. Non-inferiority will be concluded if the upper limit of the 95% confidence interval of this estimated difference remains below the stated non-inferiority margin of 3.0%. When non-inferiority has been demonstrated, the costs and effects will be expressed in the savings per percent weight reduction/patients who successfully lost weight, by means of a probabilistic model, so that the likely skewed data on costs can be considered.

For the secondary analyses of the efficacy outcomes cardiovascular risk factors (lipids), quality of life (EQ-5L), patient satisfaction (DTSQ), attrition (drop-out), and compliance (log-on information and kcal intake vs advised), we will use analysis of covariance (ANCOVA) models. The dependent variable in these models will be the outcome at T1, T2, or T3, and the independent variables will be treatment arm, the outcome at baseline (T0), and hospital. The secondary analyses are exploratory in nature as the power calculation is based on the primary outcome.

### Interim analyses {21b}

No interim analyses will be performed.

### Methods for additional analyses (e.g. subgroup analyses) {20b}

To test whether the treatment effect differs by sex and ethnicity (Dutch versus other origin), we will perform secondary analyses where, in two separate models, the three-way interaction effect of sex, treatment, and time point and the three-way interaction effect of ethnicity, treatment, and time point are respectively added to the model of the primary analysis. These models will then be used to test whether the effect of treatment on relative weight change differs by sex/ethnicity, i.e. with null hypothesis H0: relative weight change at T3 in the treatment group among men—relative weight change at T3 in the control group among men—relative weight change at T3 in the treatment group among women + relative weight change at T3 in the control group among women = 0, which can be tested using an *F* test.

### Methods in analysis to handle protocol non-adherence and any statistical methods to handle missing data {20c}

Data will be analysed based on the intention-to-treat principle. The general linear model takes drop out under the missing at random assumption into account.

### Plans to give access to the full protocol, participant-level data, and statistical code {31c}

The results of this study will be disclosed unreservedly. We will register the study before start in the Dutch Trial Registry. It is intended to publish the results as soon as possible after completion of the sample analyses and data evaluation in an appropriate peer-reviewed scientific journal. Data will be made available to other researchers upon reasonable request.

## Oversight and monitoring

### Composition of the coordinating centre and trial steering committee {5d}

The coordinating study centre is the University Medical centre, Erasmus MC in Rotterdam, Department of Dietetics, Rotterdam, The Netherlands. The trial steering committee (TSC) consists of the principal investigator, the executive investigator, the participating dietitian, and the secretariat. The TSC is responsible for all aspects of study planning and will be responsible for randomisation, data registration, data management, and biostatistics. Supervision of the study will be done by the designated trial monitor who also works at the University Medical centre, Erasmus MC in Rotterdam.

### Composition of the data monitoring committee, its role and reporting structure {21a}

Because of the low risk associated with the intervention (eHealth application), no DSMB or safety committee will be established.

### Adverse event reporting and harms {22}

Adverse events are defined as any undesirable experience occurring to a subject during the study, whether considered related to the investigational product/study procedure/the experimental intervention. All adverse events reported spontaneously by the subject or observed by the investigator or his staff will be recorded.

A serious adverse event is any untoward medical occurrence or effect that.

-Results in death;
-Is life threatening (at the time of the event);
-Requires hospitalisation or prolongation of existing inpatients’ hospitalisation;
-Results in persistent or significant disability or incapacity;
-Any other important medical event that did not result in any of the outcomes listed above due to medical or surgical intervention but could have been based upon appropriate judgement by the investigator.

An elective hospital admission will not be considered as a serious adverse event.

The investigator or anyone from the medical staff to whom the adverse event is reported will immediately inform the principal investigator who in his turn will inform the sponsor (head of the Department of Internal Medicine).

The investigator will report all SAEs to the sponsor without undue delay after obtaining knowledge of the events.

The sponsor will report the SAEs through the web portal ToetsingOnline to the accredited METC that approved the protocol, within 7 days of first knowledge for SAEs that result in death or are life threatening followed by a period of maximum of 8 days to complete the initial preliminary report. All other SAEs will be reported within a period of maximum 15 days after the sponsor has first knowledge of the serious adverse events.

All AEs will be followed until they have abated or until a stable situation has been reached. Depending on the event, follow-up may require additional tests or medical procedures as indicated and/or referral to the general physician or a medical specialist.

SAEs need to be reported till end of study within the Netherlands, as defined in the protocol.

[data safety monitoring board (DSMB)/safety committee].

### Frequency and plans for auditing trial conduct {23}

Based on the estimated risk (negligible), we opt for minimal monitoring based on the Erasmus Medical Centre guidelines (in Dutch: Richtlijnen voor on-site monitoring in relatie tot het ingeschatte risico van de studie. Versie Erasmus Medical Centre 15 november 2012 (gebaseerd op “NFU Kwaliteitsborging mensgebonden onderzoek 2.0/okt.2012)). Monitoring will take place once yearly and will be performed by an independent researcher of the Department of Internal Medicine.

### Plans for communicating important protocol amendments to relevant parties (e.g. trial participants, ethical committees) {25}

Amendments are changes made to the research after a favourable opinion by the accredited METC has been given. All amendments will be notified to the METC that gave a favourable opinion. After the METC approves the amendment, patients already participating will be notified of the changes that will affect them.

### Dissemination plans {31a}

The sponsor/investigator will submit a summary of the progress of the study to the accredited METC once a year. Information will be provided on the date of inclusion of the first subject, numbers of subjects included and numbers of subjects that have completed the study, serious adverse events/serious adverse reactions, other problems, and amendments.

The end results of this study will be disclosed unreservedly. We will register the study before start in the Dutch Trial Registry. It is intended to publish the results as soon as possible after completion of the sample analyses and data evaluation in an appropriate peer-reviewed scientific journal.

## Discussion

Adequate weight management in patients with T2D is critical to reduce associated morbidity and mortality. Current dietary care models for T2D patients are often resource intensive. With this study, we expect to answer the question whether eHealth in combination with face-to-face coaching is as effective as face-to-face coaching on reducing weight within a VLED program. This is essential given the need for more efficient work in this patient group. Previous research on this subject is not conclusive about the effects of the use of interactive eHealth on weight reduction within a diet program in patients with T2D, and the combination of face-to-face with eHealth support is scarce to date [33–36]. Expected barrier for the study is lagging patient inclusion due to COVID 19 restrictions.

By using an RCT design, we try to minimise the influence of confounders. Nevertheless, our study has some limitations: for obvious reasons, we could not blind the patient and the dietitian to the intervention. Furthermore, we use intermediate outcome measures such as weight and HbA1c because it was not feasible in terms of patient numbers and length of follow-up to report on hard endpoints like morbidity or mortality. The strength of our study is the use of three study sites in all settings of health care, making the results widely generalizable.

The findings of this study, if successful, will lead to more knowledge about the use of eHealth in lifestyle treatment for patients with T2D, more specifically on the combination of face-to-face and eHealth support. It will also potentially lead to the implementation of this eHealth, evidence-based intervention for patients with T2D. In addition, policy makers will be better informed about using eHealth for population health management.

### Trial status

Protocol version number 4, November 12, 2020.

Start recruitment: April 1, 2021.

End of recruitment (estimated): July 1, 2023.

## Data Availability

The datasets generated and/or analysed during the current study will be available from the corresponding author on reasonable request.

## Abbreviations

AE: Adverse event
CCMO: Central Committee on Research Involving Human Subjects; in Dutch: Centrale Commissie Mensgebonden Onderzoek
DSMB: Data safety monitoring board
EU: European Union
IC: Informed consent
METC: Medical research ethics committee (MREC); in Dutch: medisch-ethische toetsingscommissie (METC)
POWER: Prevention Of Weight Regain trial (referring to the diet intervention studied in this trial)
(S)AE: (Serious) adverse event
Sponsor: The sponsor is the party that commissions the organisation or performance of the research, for example a pharmaceutical company, academic hospital, scientific organisation, or investigator. A party that provides funding for a study but does not commission it is not regarded as the sponsor but referred to as a subsidising party
T2D: Type 2 diabetes
VLED: Very low energy diet
WMO: Medical Research Involving Human Subjects Act; in Dutch: Wet Medisch-wetenschappelijk Onderzoek met Mensen
